# Designing Bifunctional Electrocatalysts Based on Complex Cobalt-Sulfo-Boride Compound for High-Current-Density Alkaline Water Electrolysis

**DOI:** 10.1021/acs.energyfuels.4c03171

**Published:** 2024-09-25

**Authors:** Akash Suryawanshi, Riya Alice B. John, Aniruddha Bhide, Suraj Gupta, Matjaž Spreitzer, Rupali Patel, Rohan Fernandes, Nainesh Patel

**Affiliations:** † Department of Physics and Electronics, Christ University, Bengaluru 560029, India; ‡ Advanced Materials Department, Jožef Stefan Institute, Jamova 39, 1000 Ljubljana, Slovenia

## Abstract

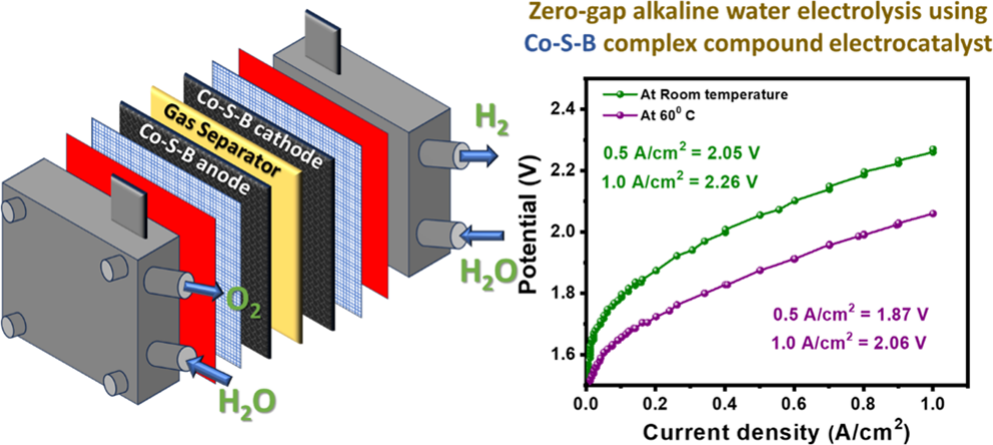

In the quest to harness renewable energy sources for green hydrogen production, alkaline water electrolysis has emerged as a pivotal technology. Enhancing the reaction rates of overall water electrolysis and streamlining electrode manufacturing necessitate the development of bifunctional and cost-effective electrocatalysts. With this aim, a complex compound electrocatalyst in the form of cobalt–sulfo–boride (Co–S–B) was fabricated using a simple chemical reduction method and tested for overall alkaline water electrolysis. A nanocrystalline form of Co–S–B displayed a combination of porous and nanoflake-like morphology with a high surface area. In comparison to Co–B and Co–S, the Co–S–B electrocatalyst exhibits better bifunctional characteristics requiring lower overpotentials of 144 mV for hydrogen evolution reaction and 280 mV for oxygen evolution reaction to achieve 10 mA/cm^2^ in an alkaline electrolyte. The improved Co–S–B performance is attributed to the synergistic effect of sulfur and boron on cobalt, which was experimentally confirmed through various material characterization tools. Tafel slope, electrochemical surface area, turnover frequency, and charge transfer resistance further endorse the active nature of the Co–S–B electrocatalyst. The robustness of the developed electrocatalyst was validated through a 50 h chronoamperometric stability test, along with a recyclability test involving 10,000 cycles of linear sweep voltammetry. Furthermore, Co–S–B was tested in an alkaline zero-gap water electrolyzer, reaching 1 A/cm^2^ at 2.06 V and 60 °C. The significant activity and stability demonstrated by the cobalt-sulfo-boride compound render it as a promising and cost-effective electrode material for commercial alkaline water electrolyzers.

## 1 Introduction

The pressing need to develop sustainable energy sources in the face of climate change and depleting fossil fuel reserves has encouraged researchers to explore innovative solutions that can revolutionize our energy landscape.^1^ One such solution, the electrolysis of alkaline water for green hydrogen (H_2_) production from intermittent energy sources, offers a promising avenue for the efficient storage and conversion of renewable energy.^2^ H_2_ is also widely used in the industrial processes of ammonia synthesis, petroleum refining, and electricity generation using fuel cells.^3−5^ Alkaline water electrolysis has attracted extensive attention, but highly efficient and stable catalysts are required to promote the sluggish kinetics of the two half-reactions, i.e., hydrogen evolution reaction (HER) and oxygen evolution reaction (OER).^6^ The traditional alkaline water electrolyzers (AWEs) face challenges like low current density (<0.25 A cm^–2^) with an efficiency of only up to 60% and incompatibility issues with dynamic input from renewable energy sources. On the other hand, proton exchange membrane water electrolyzers (PEMWEs) solve the problem related to low current density and efficiency, but this high performance is offset by reliance on expensive noble-metal catalysts such as platinum and iridium.^2,7,^ Anion exchange membrane water electrolyzer (AEMWE) technology integrates the benefits of both AWEs and PEMWEs. It provides highly efficient water electrolysis under dynamic operational conditions while enabling the use of earth-abundant transition metal catalysts for both the cathode and the anode. Despite these advantages, electrodes in the AEMWE stack still represent a considerable share of the overall electrolyzer cost.^9^ As a result, a significant amount of AEMWE research is focused on the development of new, efficient, and cost-effective catalysts for the electrodes that can function well in an alkaline environment, leading to a reduction in expenses and improved performance.^10,11^

Until now, a vast majority of earth-abundant transition metal compounds such as phosphides,^12−14^ carbides,^15,16^ sulfides,^17,18^ selenides,^19,20^ nitrides,^21,22^ and borides^23−26^ have been explored as promising electrode materials for alkaline water electrolysis. The non-metal/metalloid component in each of these compounds has played a unique role in enhancing the electrochemical properties of the transition metals. For example, metal nitrides have garnered substantial interest owing to their distinctive metal-like properties, including enhanced d-electron density on metal sites upon nitrogen incorporation, thereby mirroring the electronic structure of noble metals such as Pd and Pt.^27^ Similarly, borides and phosphides modulate the electrons surrounding the metal and non-metal sites, respectively, making them active for the adsorption of the hydrogen species during HER while acting as the pre-catalyst for the generation of the oxy-hydroxide species for the OER.^28^ Metal sulfides offer appealing attributes such as chemical stability by preventing metal particles from aggregation or dissolution during the reaction, as demonstrated by Co_9_S_8_ and Co_3_S_4_ nanostructures.^29,30^ Moreover, sulfur can modify the surface properties, such as increasing the surface area and introducing active sites for catalysis. Significant improvement has also been achieved using bimetallic transition metal sulfide^31^ and bimetallic transition metal boride compounds^32^ deposited on conductive nickel foam as a bifunctional electrocatalyst for overall water splitting. While each of these compounds offer distinct benefits, forming a complex compound by integrating two or more non-metals/metalloids with a transition metal presents a compelling path to enhance the catalytic activity further, leveraging their synergistic effects. Such investigations have rarely been undertaken, and only a few research groups have explored the combined effect of phosphides and borides^33,34^ for water splitting and sulfides and borides for supercapacitor applications.^35^ However, the sulfide-boride combination for water splitting application has only been elucidated in the work of Hui et al. through the development of a Ni–S–B coating.^36^ This coating demonstrated remarkable HER activity with an impressively low onset overpotential of 27 mV under alkaline conditions. This underscores the untapped potential of such complex compound electrocatalysts in greatly enhancing the electrocatalytic performance.

Building upon these studies, the present research reports cobalt-sulfo-boride (Co–S–B) catalyst for the first time for alkaline water splitting. Through experimental screening, an optimized composition of Co–S–B, which exhibits superior bifunctional activity for water splitting under alkaline conditions, was developed. The role of Co, S, and B in promoting the catalytic activity of Co–S–B was determined with the help of pre- and post-activity investigation of the material properties. This study presents Co–S–B as a robust catalyst for industrial alkaline water splitting and exemplifies the possibility of developing new complex compound electrocatalysts, laying the foundation for future research in this exciting new class of materials.

## 2 Experimental Method

### 2.1 Chemicals and Reagents

Cobalt chloride hexahydrate (CoCl_2_·6H_2_O, 99% Researchlab), sodium sulfide nonahydrate (Na_2_S·9H_2_O, 99% Anmol Chemicals), sodium borohydride (NaBH_4_, 98% Researchlab), ethanol (99%, Northman), Nafion (20%, Sigma-Aldrich), ammonium chloride (NH_4_Cl, 99%, Researchlab), ammonium hydroxide (NH_3_OH, 25%, Researchlab), potassium hydroxide pellets (KOH, 99% Researchlab), sodium hydroxide pellets (NaOH, 99% Researchlab), acetone (C_3_H_6_O, 99% Researchlab), hydrochloric acid (HCl, 98%, Researchlab), and deionized (DI) water, which was used as the general-purpose solvent. Nickel foam (0.5 mm) was acquired from Dtech Solution, India.

### 2.2 Synthesis of Cobalt–Sulfo–Boride (Co–S–B) Catalyst

Co–S–B catalyst was synthesized by a facile one-step chemical reduction method. Here, a mixture of sodium borohydride (NaBH_4_) and sodium sulfide (Na_2_S·9H_2_O), as precursors for B and S, respectively, were introduced in the aqueous solution of cobalt chloride (CoCl_2_) (0.05 M) under vigorous stirring.^37^ Here, NaBH_4_ also acts as a reducing agent. The molar ratio of the reducing agent to cobalt salt was maintained three times to ensure the complete reduction of the metal cations. The mixture was continuously stirred until the effervescence ceased (about 20 min). The resulting black precipitate was filtered and then thoroughly washed using ethanol and DI water before drying in a vacuum environment. The B/S molar ratio was varied in the catalysts by altering the molar concentration of the precursors in the aqueous solution. For comparison, Co–B and Co–S catalysts were also synthesized by reducing Co salt separately with NaBH_4_ and Na_2_S·9H_2_O, respectively.

### 2.3 Materials Characterization

For structural analysis of the catalyst powders, an X-ray diffractometer (Rigaku-miniflex) using Cu Kα (1.5418 Å) radiation was used in the Bragg–Brentano configuration (θ–2θ). The morphologies and chemical composition of the as-prepared catalysts were characterized by field emission scanning electron microscopy (FE-SEM) (Thermo Scientific Apero 2S) at an acceleration voltage of 10 kV. Transmission electron microscopy (TEM) and high-resolution TEM (HRTEM) were carried out on TALOS F200S G2 with an acceleration voltage of 200 kV. The multipoint Brunauer–Emmett–Teller (BET) facility (Belsorp Mini X) was used to determine the surface area from nitrogen adsorption–desorption isotherms. The surface chemical state and atomic compositions were determined using X-ray photoelectron spectroscopy (XPS, PHI VersaProbe III instrument, AlKα-1486.6 eV). The binding energy (BE) of each element was calibrated by considering the C 1s spectrum (284.8 eV) as the reference. The Raman spectra of the catalysts were acquired by using a laser with an excitation wavelength of 532 nm in a Renishaw InVia Raman microscope. For XPS and Raman analysis of the catalysts after electrochemical testing, the powder catalyst was recovered from the surface of the glassy carbon electrode, dispersed in deionized water, filtered, and dried.

### 2.4 Preparation of Working Electrode

For the initial screening of the electrocatalysts, the as-prepared powders were deposited on a glassy carbon electrode (GCE) and used as the working electrode. For electrochemical testing, a homogeneous catalyst slurry was prepared by mixing 5 mg of catalyst powder in 1 mL of ethanol solvent, followed by 10 min dispersion in an ultrasonic bath. A separate binder solution was prepared by mixing 1 mL of ethanol and 40 μL of Nafion solution. A defined volume (20 μL) of catalyst ink and binder solution (10 μL) were drop-casted successively onto the polished surface of a 3 mm glassy carbon electrode (GCE, geometric surface area = 0.07 cm^2^). With this deposition method, the mass loading was always maintained at 0.7 mg/cm^2^. An optimized Co–S–B catalyst was deposited on porous nickel foam (Co–S–B/NF) for chronoamperometric stability, reusability, and electrolyzer tests. A modified electroless plating method was employed to deposit a Co–S–B catalyst on a Ni-foam substrate (0.5 mm thickness) with a geometrical area of 0.5 cm^2^ (henceforth denoted as Co–S–B/Ni-foam).^38^ In this process, an aqueous mixture containing cobalt chloride (CoCl_2_), ammonium chloride (NH_4_Cl), and ammonium hydroxide (NH_4_OH) was prepared as solution A, and another mixture containing a mixture of sodium borohydride (NaBH_4_) and sodium sulfide nonahydrate (Na_2_S·9H_2_O), was prepared as solution B. A piece of precleaned Ni foam (0.5 cm^2^) was immersed in solution A, followed by adding an equal amount of solution B for the reduction process. This cycle of immersion and reduction was repeated 10 times to ensure uniform catalyst deposition onto the substrate, and the resulting electrode was dried under an infrared lamp.

### 2.5 Electrochemical Measurements

For electrochemical testing, a conventional three-electrode system was utilized comprising a catalyst-coated GCE, saturated calomel electrode (SCE), and graphite electrode as working, reference, and counter electrodes, respectively. 1 M KOH aqueous solution was used as the electrolyte, and the electrochemical measurements were conducted using a potentiostat (CH 16011E). The amount of electrolyte and the distance between the electrodes were kept constant for all of the measurements. Before actual HER measurements, the surface conditioning of the cathode catalyst was carried out by performing chronoamperometry measurements until the observed current was stabilized, ensuring the removal of any surface native oxide layers. The electrolyte was continuously stirred to avoid any bubble accumulation over the electrodes.^39^ The potential measured with respect to the calomel electrode was converted to a reversible hydrogen electrode (RHE) using the Nernst equation *E* = 0.241 + (0.059*pH). Electrochemical impedance spectroscopy (EIS) was performed in the frequency range of 1 MHz–1 Hz in potentiostatic mode at constant potentials of 1.67 and −0.24 V (vs RHE) to determine the charge transfer resistance (*R*_ct_) and solution resistance (*R*_s_) during the OER and HER, respectively. *R*_s_ value obtained from EIS was used for *iR* compensation (100%). Linear sweep voltammetry (LSV) in the range of 1.0–1.5 V (vs RHE) (at 2 mV/s) was performed to investigate the pre-oxidation behavior of the catalyst. The catalyst’s electrochemical surface area (ECSA) was also determined to estimate the active surface area of the catalyst under an electrolyte environment. ECSA can be directly correlated to the double-layer capacitance (*C*_dl_) formed at the interface of the electrode and electrolyte. The *C*_dl_ capacitance was determined in the non-Faradaic region by sweeping the potential in the range of 100 mV across the open-circuit potential at varying scan rates of 20, 40, 60, 80, 100, and 120 mV/s in 1 M KOH. From these CV curves, the difference between the capacitive current (Δ*J* = *|j*_cathodic_ – *j*_anodic_|), measured at a specific potential, was plotted against the respective scan rates. The slope obtained by linear fitting is twice the value of the *C*_dl_ at the interface of the electrolyte and catalyst.^40^ Tafel slope was determined by linear fitting of the plot of log (*i*) versus overpotential (η). The turnover frequency (TOF) was calculated using a previously reported method.^41^ Using optimized Co–S–B deposited on porous nickel foam (Co–S–B/NF), the long-term stability test was performed through chronoamperometric tests at a constant overpotential of 310 mV (OER) and 174 mV (HER) for 15 h. Co–S–B/NF catalyst recycling was performed by conducting 10,000 cycles at a scan rate of 100 mV/s. The optimized catalyst was tested in 2-electrode configurations using Co–S–B/Ni-foam as cathode and anode in a conventional electrolysis cell. Potentiostatic stability measurement was performed on Co–S–B/NF in a two-electrode assembly for 50 h at a constant potential of 1.68 V in 6 M KOH. Finally, Co–S–B/NF electrodes were employed in a zero-gap configuration within a single-cell electrolyzer (5 cm^2^), where a commercial Zirfon membrane was used as the separator. Symmetric feed was used as the electrolyte (6 M KOH) was circulated through the anodic and cathodic chambers using a peristaltic pump at room temperature and 60 °C. Polarization curves were measured by sweeping the current density in the range of 0–1 A cm^–2^, while the corresponding potentials were simultaneously recorded. The voltage efficiency of the single-cell electrolyzer was calculated using the equation:  as reported by Santos et al.^42^

## 3 Results and Discussion

Co–S–B catalyst presents a unique complex compound between a transition metal (Co), a non-metal (S), and a metalloid (B). To determine the most active composition, the molar ratio of B/S was first varied from 1 to 10, keeping the Co amount constant. Based on the catalytic performance (overpotential recorded at 10 mA/cm^2^, η_10_) for HER and OER (discussed later), the optimal B/S ratio was identified as 8, which will be henceforth referred to as Co–S–B-8 and was primarily characterized along with Co–B and Co–S samples.

**Figure 1:**
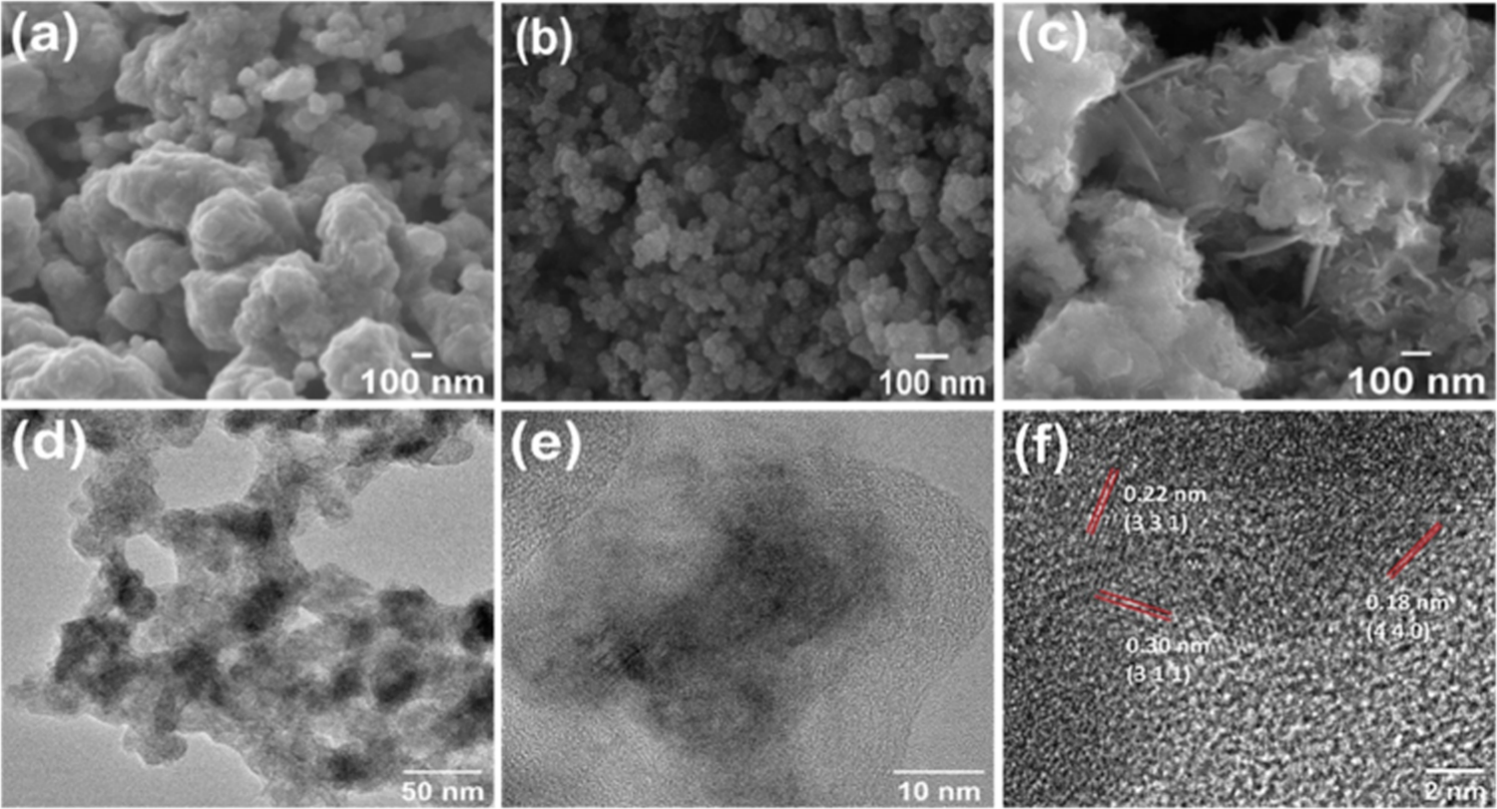
FE-SEM image of (a) Co–S, (b) Co–B, (c) Co–S–B-8; (d, e) TEM image and (f) HRTEM image of Co–S–B-8.

Based on SEM analysis, the Co–S catalyst (Figures 1a and S1a) depicts spherical particle-like morphology with an average size of 290 (±77.57) nm (Figure S2a). A similar particle-like morphology is observed for the Co–B catalyst (Figure 1b) but with a much smaller average particle size of 45 ± 9.77 nm (Figure S2b). The difference in particle sizes can be attributed to the difference in the reducing agents used during the synthesis. On the contrary, when both the precursors of S and B are used in tandem, a complete morphological transformation is observed for the Co–S–B-8 catalyst, where a porous and fibrous wool-like morphology along with 2D nanoflakes were observed (Figures 1c and S1b). TEM images also confirm the porous nature of the Co–S–B-8 particles (Figure 1d), along with the presence of 2D nanoflakes (Figure 1e). The HRTEM images of Co–S–B-8 (Figure 1f) show distinct lattice fringes with an interplanar spacing of 0.22, 0.30, and 0.18 nm corresponding to (331), (311), and (440) planes of Co_9_S_8_ phase, respectively.^43,44^ These fringes arise from small nano-crystalline domains which seem to be embedded in an amorphous matrix. Elemental mapping obtained using the EDS profile (Figure S3) substantiates the presence and uniform distribution of all of the elements in the Co–S–B-8 catalyst. Boron was not detected convincingly due to the instrument’s lower detection limit, but the presence of boron was confirmed later through XPS.

**Figure 2:**
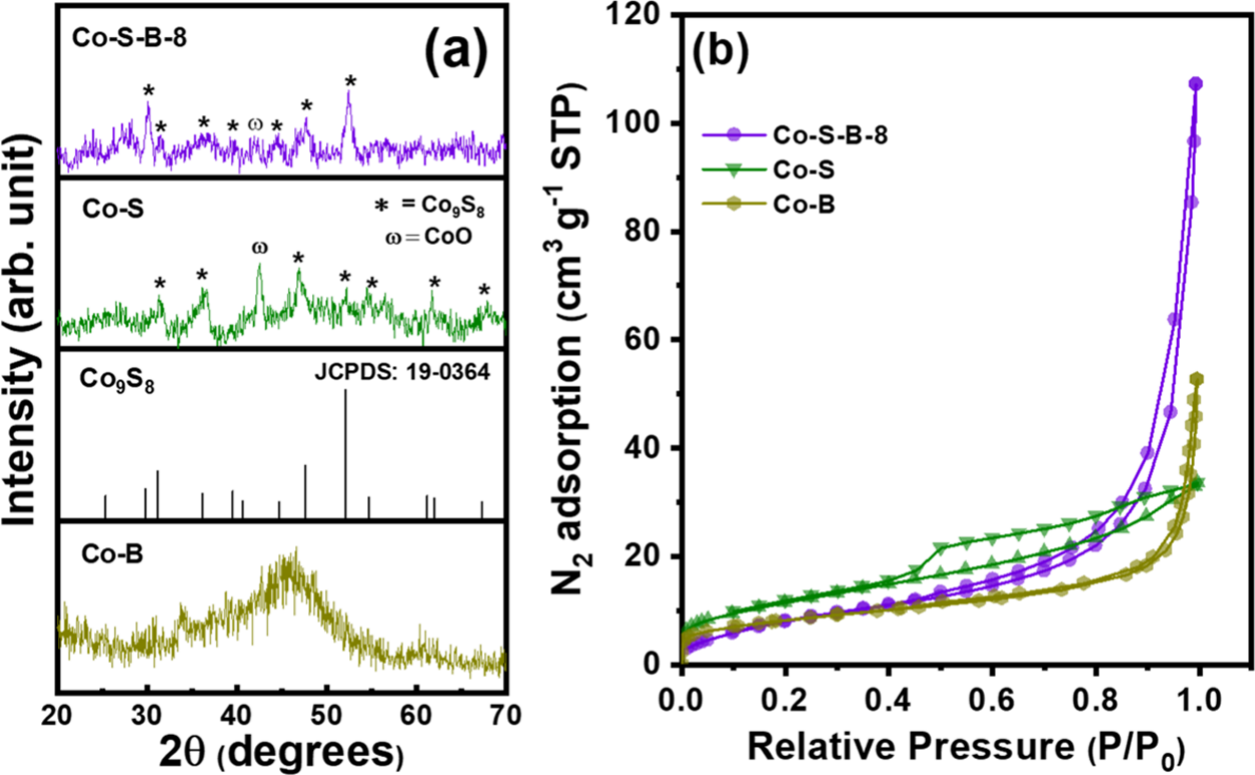
(a) XRD pattern and (b) nitrogen adsorption–desorption isotherms for Co–S, Co–B, and Co–S–B-8.

X-ray diffractogram (Figure 2a) shows a broad hump centered at 2θ = 45°, affirming the amorphous phase of the Co–B catalyst, aligning well with the previous reports.^45^ The Co–S and Co–S–B-8 catalysts exhibit distinct peaks at 30.1, 31.4, 36.2, 39.6, 44.6, 47.7, and 52.4° primarily assigned to crystallographic planes (311), (222), (400), (331), (422), (511), and (440) of the Co_9_S_8_ (JCPDS = 19-0364) phase. A single peak at 45.5° corresponding to CoO (JCPDS = 78-0431) phase is also noticed, mainly in Co–S. The weak intensity of these peaks suggests formation of small polycrystalline domains, within an amorphous phase, which was also observed from HRTEM image. The specific surface area was determined as 28.9, 41.7, and 31.7 m^2^ g^–1^ for Co–B, Co–S, and Co–S–B-8 catalysts, respectively, using BET analysis (Figure 2b). The isotherm for Co–S exhibits a type IV isotherm with an H3-type hysteresis loop, indicative of the presence of mesoporous particles. This finding suggests that Co–S particles are mesoporous in nature, although they are larger in size, thus giving rise to the highest surface area among all of the catalysts. On the other hand, Co–B and Co–S–B-8 display type III isotherm, suggesting the presence of macropores formed between nanoparticles. The marginally higher surface area of Co–S–B-8 compared to Co–B could be due to the mixed nanoflake and fibrous wool-like morphology.

**Figure 3:**
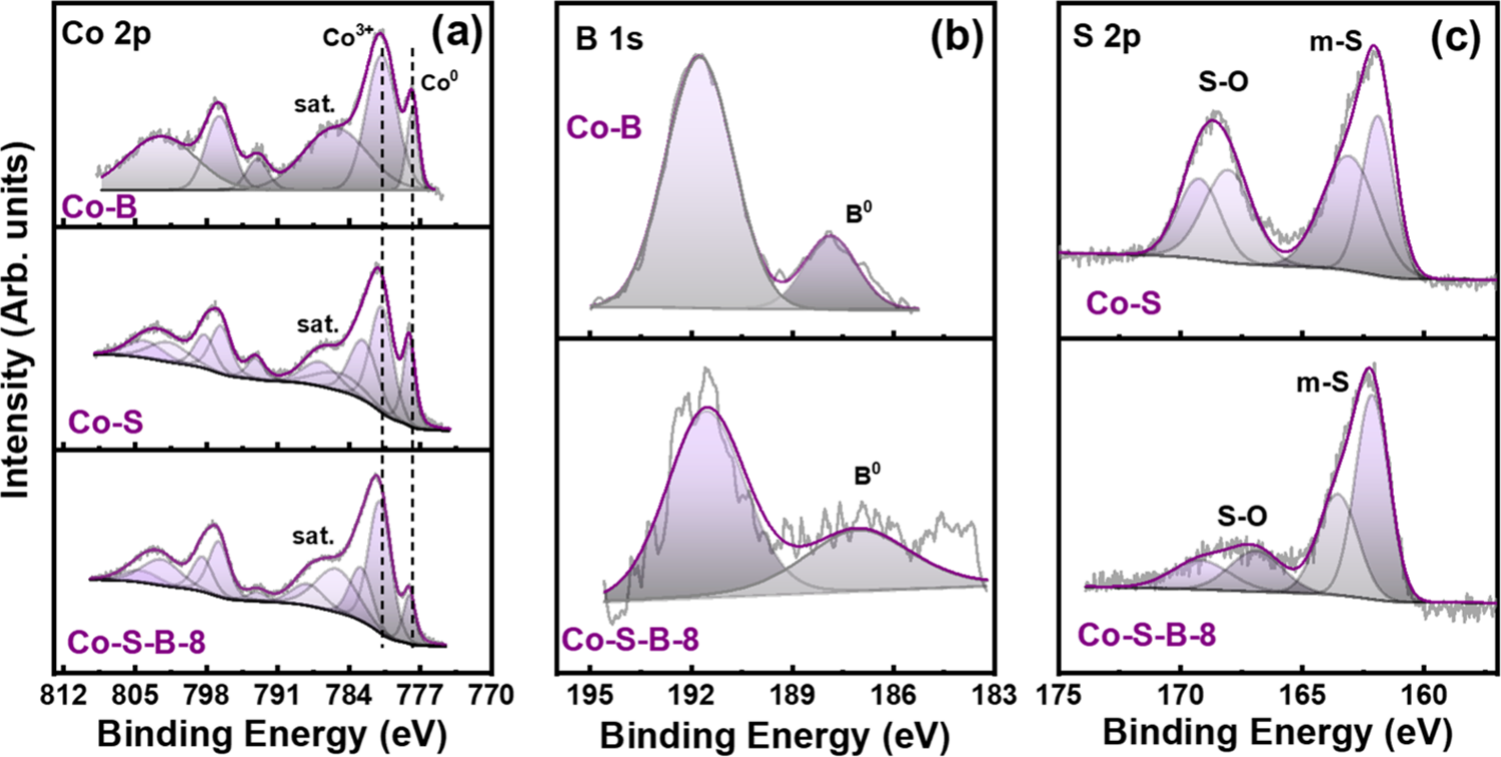
XPS spectra for (a) Co 2p, (b) B 1s, and (c) S 2p level in Co–B, Co–S, and Co–S–B-8.

Detailed information about the surface states of the catalysts was obtained using XPS (Figure 3a–c). For Co–S–B-8, the broad peak in the Co 2p level (Figure 3a) was deconvoluted into multiple peaks with binding energy (BE) centered at 777.6 eV corresponding to elemental cobalt (Co^0^) and at 780.8 and 782.7 eV attributed to trivalent (Co^3+^) and divalent (Co^2+^) cobalt oxide, respectively. Similarly, for binary compounds of Co–S and Co–B, metallic cobalt peaks are detected at 778.2 and 777.7 eV, respectively. When compared to a reference cobalt metal (778.2 eV), a negative shift of about 0.5 and 0.6 eV is seen in Co–B and Co–S–B-8, which is typical of metal boride compounds. The presence of oxidized cobalt (Co^2+^ and Co^3+^) is attributed to ex situ catalyst preparation and its exposure to the ambient atmosphere during the catalyst transfer to the measurement chamber. The satellite peaks of both oxidation states are identified in the range of 785.0–787.0 eV. In the B 1s state (Figure 3b), both Co–B and Co–S–B-8 show a peak corresponding to elemental boron at 187.9 and 187.7 eV, respectively. However, the BE of this peak is positively shifted by 0.8 eV for Co–B and 0.6 eV for Co–S–B-8 when compared to that of pure boron (187.1 eV), indicating partial transfer of electrons from boron. This makes the metal sites highly active by enriching them with electrons and filling their vacant d-orbitals, as also evidenced by the negative shift in the BE of metallic Co.^46,47^ The peaks corresponding to surface oxidized boron (B_2_O_3_) are also observed at 191.7 and 191.9 eV for both catalysts. The two broad peaks in the S 2p level (Figure 3c) of Co–S and Co–S–B-8 were deconvoluted into various peaks. Among them, the two lower-energy peaks (162.1 and 163.5 eV) correspond to the metal–sulfide (m–S) bond, while the higher-energy peaks (166.9 and 169.2 eV) suggest surface oxidation and formation of sulfites.

**Figure 4:**
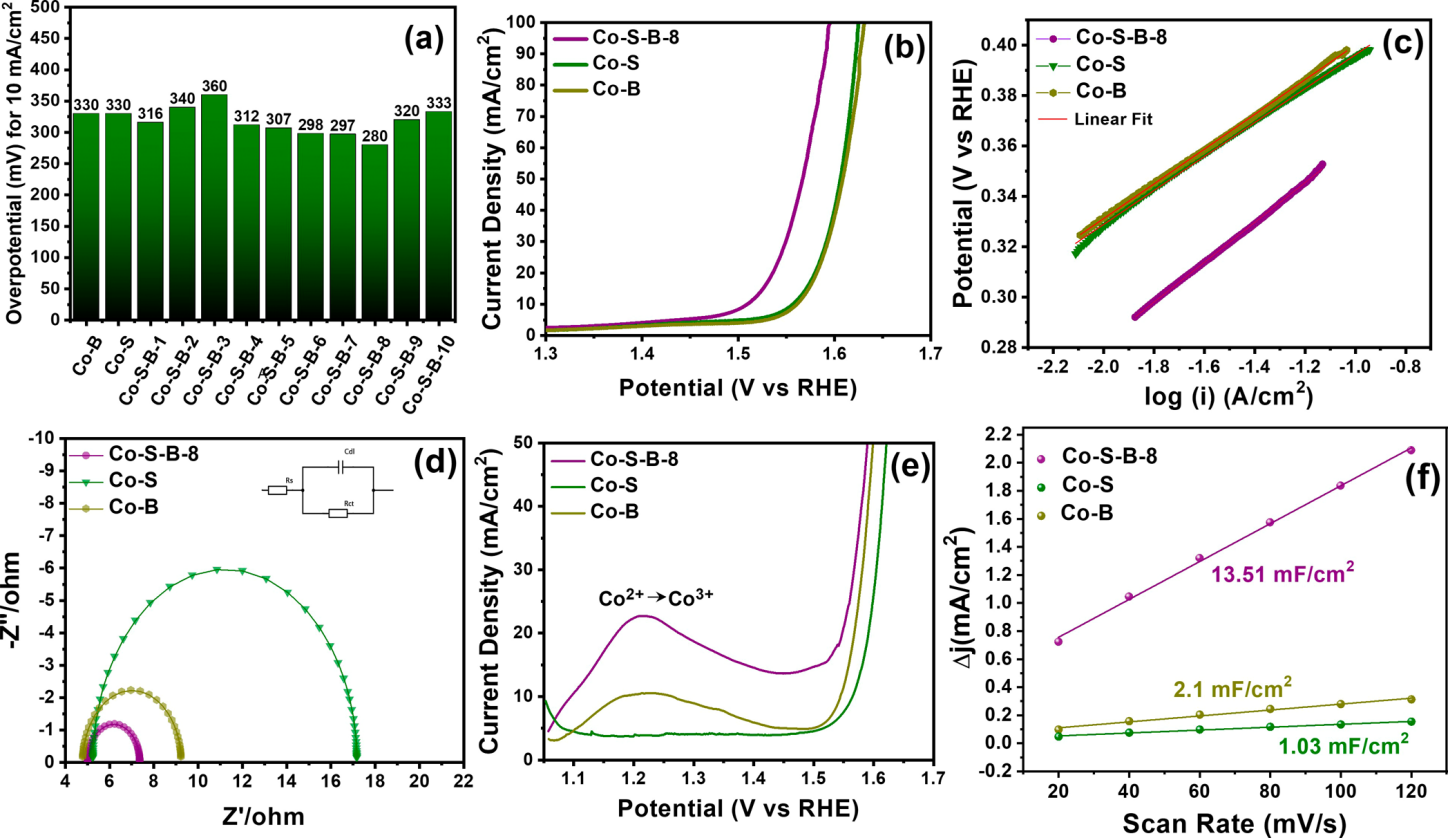
(a) Comparison plot of overpotential value for the OER recorded at 10 mA/cm^2^ for Co–S–B catalysts of different B/S ratios, (b) Linear polarization curve (*iR* compensated) at 10 mV/s, (c) Tafel plot, (d) Nyquist plot, and (e) linear polarization curve (*iR* compensated) in the pre-OER region for Co–S–B-8, Co–B, and Co–S catalysts in 1 M KOH at 2 mV/s. (f) Plot representing the difference in cathodic and anodic current densities at different scan rates to determine the double-layer capacitance (*C*_dl_) of Co–S–B-8, Co–B, and Co–S catalyst.

To determine the optimal composition of the Co–S–B catalyst for electrochemical water splitting, the B/S ratio was varied from 1 to 10 and the OER activity was measured for all of the ratios (Figures 4a and S4a). Notably, the lowest overpotential was observed for the B/S ratio of 8, while it also showed the lowest charge transfer resistance (*R*_ct_) (Figure S4b). Linear polarization curves (Figure 4b) reveal that Co–S–B-8 exhibits the lowest OER overpotential (η_10_) of 280 mV when also compared to the control samples of Co–B (330 mV), Co–S (330 mV), and RuO_2_ (370 mV). The electrocatalytic activity was repeated several times to reproduce and confirm the results (Figure S5). Moreover, the Tafel slope values of Co–S–B-8 (79 mV/dec), Co–B (68 mV/dec), and Co–S (67 mV/dec) catalysts are in the same range, suggesting similar kinetics during the OER (Figure 4c). The Nyquist plot (Figure 4d) demonstrates that Co–S–B-8 has considerably lower *R*_ct_ (2.36 Ω) compared to Co–B (4.44 Ω) and Co–S (11.93 Ω). Another notable parameter for the OER mechanism is the formation of surface-active –OOH* species, which can be probed from the pre-oxidation peak formation. The peak observed in the pre-oxidation range of 1.18–1.24 V (Figure 4e) corresponds to the Co^2+^ to Co^3+^ conversion, leading to the formation of Co-OOH* active species for the OER. This peak was predominantly evident in boron-containing compounds of Co–B and Co–S–B-8 but was absent for the Co–S electrocatalyst. The observed behavior is consistent with the pre-catalyst theory,^48^ which suggests that metal borides act as the pre-catalyst and undergo surface reconstruction during OER to form oxy-hydroxide species. Moreover, the peak intensity suggests the quantity of active Co-OOH* sites formed on the surface. Therefore, the exceptional OER activity of the Co–S–B-8 electrocatalyst in an alkaline environment can be attributed to the peak’s maximum intensity during pre-oxidation. The formation of active sites was further substantiated by calculating the electrochemical surface area via double-layer capacitance (*C*_dl_). The *C*_dl_ values of 13.51 mF cm^–2^, determined from CV scans (Figure S6), are highest for Co–S–B-8, which is about 6.4 and 13.1 times compared to Co–B (1.62 mF cm^–2^) and Co–S (1.03 mF cm^–2^) (Figure 4f). This highlights the significant number of electrochemically active sites available, resulting in enhanced catalytic activity for Co–S–B-8 compared with other developed catalysts. Turnover frequency (TOF) values of 0.3074, 0.2691, and 0.3856 s^–1^ were obtained (at 400 mV overpotential) for Co–B, Co–S, and Co–S–B-8, respectively. The superior OER rates for Co–S–B-8 are attributed to the formation of a large number of Co-OOH* active species, which increases the electrochemical surface area and reduces the charge transfer resistance across the electrolyte and electrode interface.

**Figure 5:**
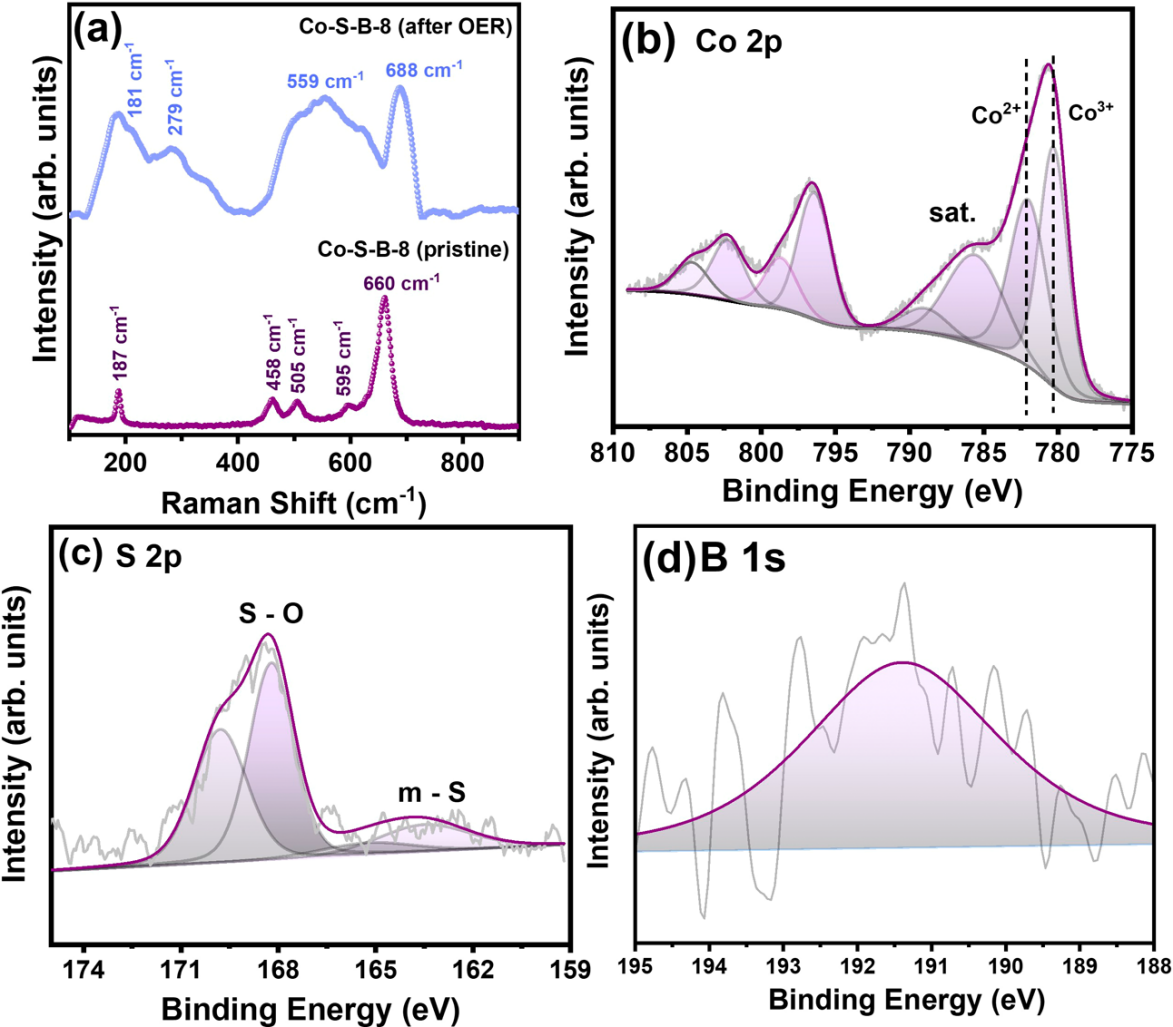
(a) Raman spectra of Co–S–B-8 before and after OER in 1 M KOH. Post-OER high-resolution XPS spectra of (b) Co 2p, (c) S 2p, and (d) B 1s states of Co–S–B-8 tested in 1 M KOH.

The chemical changes on the surface of the solid Co–S–B-8 catalyst post-OER were investigated by using Raman spectroscopy and XPS (Figure 5). In pristine Co–S–B-8 catalyst, the peaks (187, 458, 505, 595, and 660 cm^–1^) related to surface oxides (Co_3_O_4_ phase) of cobalt are clearly visible (Figure 5a), which is inevitable when the catalyst is exposed to air.^49^ These oxide layers do not pose any undesired hindrance to the catalytic activity, and during OER, they transform to CoOOH (559 and 688 cm^–1^) and Co(OH)_2_ species (181 and 279 cm^–1^), which are vital steps in alkaline OER. The predominant peak shift in the post-OER catalyst (688 cm^–1^) compared to Co–S–B (pristine) (660 cm^–1^) shows that Co–S–B acts as a pre-catalyst while the formation of active CoOOH species takes place during OER to facilitate the reaction kinetics. Further evidence of CoOOH formation on the catalyst surface post-OER is provided by the evolution of two new peaks at 780.3 and 781.5 eV, assigned to γ-CoOOH and Co(OH)_2_ species, in Co 2p level of XPS spectra (Figure 5b).^50^ The doublet peaks corresponding to elemental sulfur (Figure 5c) are transformed to sulfur-oxide post-OER, as seen from the higher oxidation peak. On the contrary, the visibility of boron is marginal in the post-OER spectra of B 1s level (Figure 5d), thus suggesting that the boron might be etched out from the surface during the reaction. The etching of boron was observed in previous reports^51^ and is said to be responsible for improving the electrochemical surface area, which is also evident in our case (Figure 4f).

**Figure 6:**
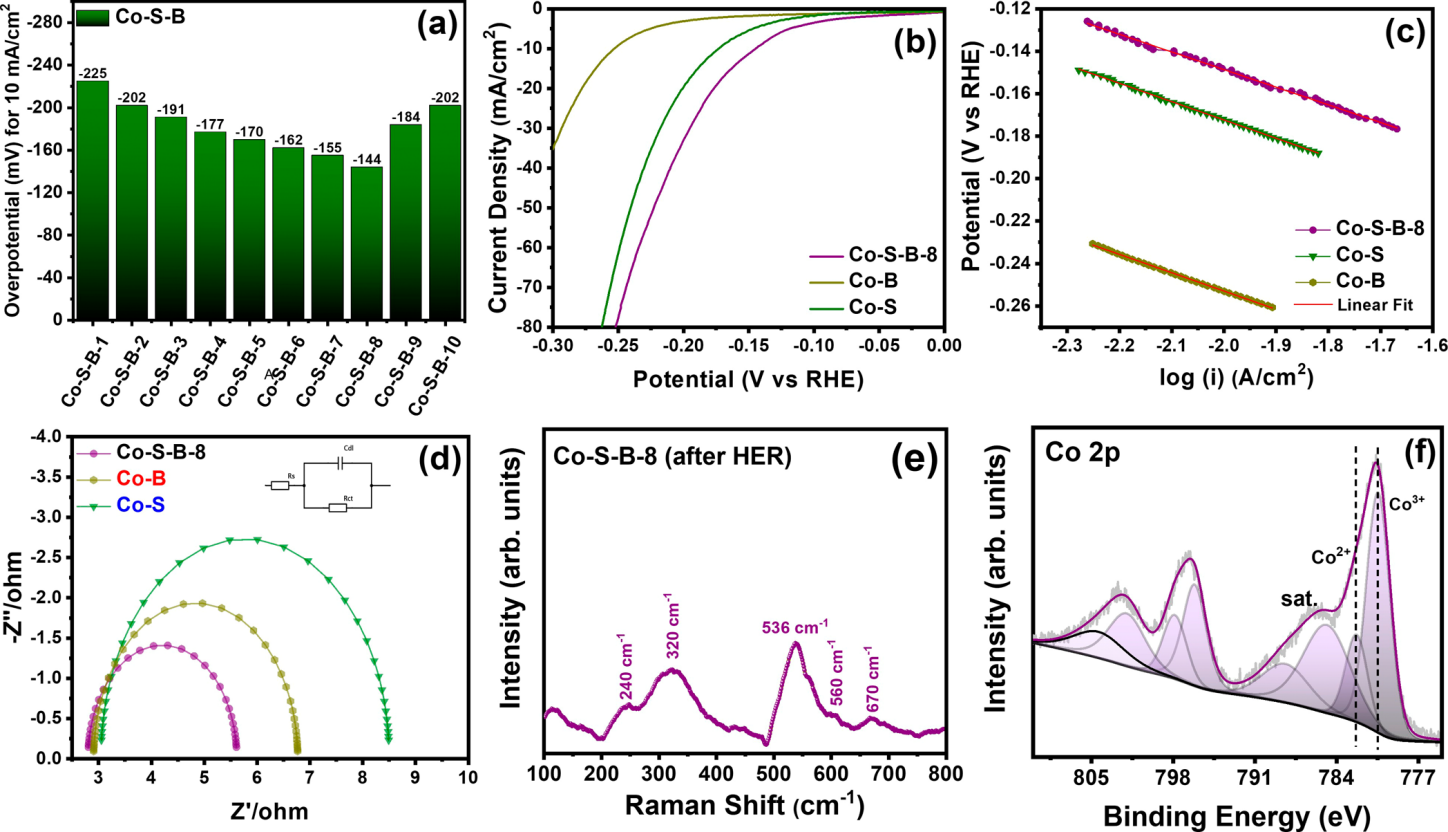
(a) Comparison plot of overpotential value for HER recorded at 10 mA/cm^2^ for Co–S–B catalysts with different B/S ratios. (b) Linear polarization curve (*iR* compensated), (c) Tafel plot, and (d) Nyquist plot for HER using Co–S–B-8, Co–B, and Co–S catalyst in 1 M KOH, (e) Raman spectra, and (f) XPS spectra for Co 2p states of Co–S–B-8 post-HER.

To elucidate the bifunctional behavior, the developed electrocatalysts were also investigated for the HER. Figure 6a compares the HER activity for Co–S–B catalysts with different B/S ratios. Similar to OER, even in HER, the highest catalytic performance was obtained for B/S = 8 (Figure S7a and Table S1), which is again due to its lowest charge transfer resistance (Figure S7b). Co–S–B-8 exhibits the overpotential (η_10_) value of 144 mV. In comparison, Co–S and Co–B necessitate higher overpotentials of 172 and 253 mV, respectively, while noble metal Pt shows the lowest overpotential of 35 mV. The comparison between Co–S, Co–B, and Co–S–B-8 highlights the pivotal role of the complex compound during HER (Figure 6b). The Tafel slope for Co–S–B-8, Co–B, and Co–S was calculated as 83, 86, and 86 mV/dec, respectively, concluding that the HER proceeds through the Volmer–Heyrovsky mechanism for all three catalysts (Figure 6c). The charge transfer behavior at the electrode/electrolyte interface was investigated by fitting the Nyquist plot (Figure 6d) using an equivalent circuit (inset of Figure 6d). The *R*_ct_ determined for Co–S–B-8 (2.8 Ω) shows a lower value than those of Co–S (5.4 Ω) and Co–B (3.8 Ω). For HER, the TOF values of 0.0337, 0.0440, and 0.0700 s^–1^ were computed for Co–B, Co–S, and Co–S–B-8, respectively, at an overpotential of 200 mV. The formation of complex compound not only increases the number of electrochemically active sites but also improves the intrinsic activity at each site, which is largely due to the synergic effect created by the presence of both B and S in the same compound. The chemical changes on the surface of the Co–S–B-8 catalyst post-HER were investigated by Raman spectroscopy (Figure 6e), where surface oxides are mainly transformed into Co(OH)_2_.^52^ The transformation of the surface oxides into Co(OH)_2_ was also verified by XPS analysis of the Co 2p_3/2_ level after HER (Figure 6f). The post-HER XPS spectra shows that both boron and sulfur are in oxidized states (Figure S8).

**Figure 7:**
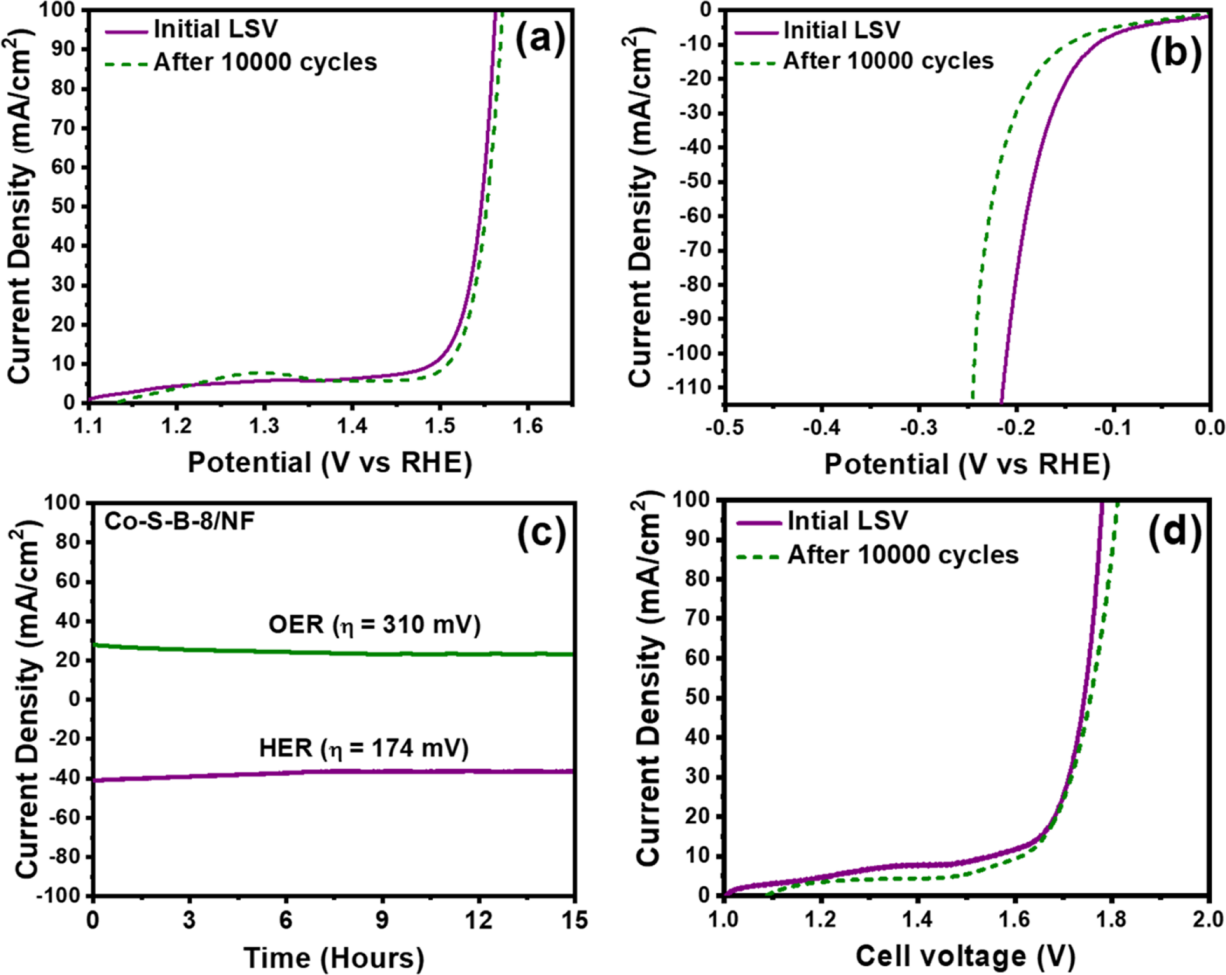
Linear polarization curve of Co–S–B-8 on Nickel foam in 1 M KOH before and after 10,000 cycles for (a) OER and (b) HER. (c) Chronoamperometric tests recorded for 15 h at a constant overpotential of 310 mV and −174 mV for OER and HER respectively. (d) Polarization curve obtained for a two-electrode configuration (Co–S–B-8/NF || Co–S–B-8/NF) before and after 10,000 cycles in 1 M KOH.

Considering commendable catalytic activities, the Co–S–B-8 catalyst was deposited onto a porous nickel foam (NF) substrate via an electroless deposition technique.^38^ This strategy resulted in notably higher catalytic activity of Co–S–B-8, requiring 330 and 198 mV for the OER and HER, respectively, to achieve 100 mA/cm^2^ (Figure 7a,b). The obtained results are attributed to the enlarged physical surface area and the highly conductive substrate provided by the porous nickel foam. A critical assessment of the catalyst reusability was carried out by measuring the Co–S–B-8/NF catalyst for 10,000 cycles of the OER and HER. The anodic current exhibited a mere 2% decrease after 10,000 cycles, suggesting exceptional reusability of Co–S–B-8 for the OER (Figure 7a). On the other hand, after 10,000 cycles of HER, the catalyst showed a decline of 14% (Figure 7b). Stability evaluations for both HER and OER were executed through chronoamperometric tests (Figure 7c) at constant overpotentials of 174 and 310 mV, respectively, for 15 h, exhibiting considerable stability. Considering the superior bifunctional catalytic activity of the Co–S–B-8/NF electrocatalyst, further investigations were performed by integrating the catalyst in a two-electrode assembly immersed in 1 M KOH. Notably, the Co–S–B-8/NF || Co–S–B-8/NF system (Figure 7d) required an overall cell voltage of 1.77 V to reach 100 mA/cm^2^, which was maintained even after 10,000 cycles of rigorous testing. The catalytic activity was sustained with minimal degradation even at higher current densities. Faradaic efficiency measurements for the HER and the OER were conducted for the optimized Co–S–B-8/NF || Co–S–B-8/NF system using the water displacement method in a Hoffman apparatus. The Faradaic efficiency was determined by measuring the volume of the evolved gases (H_2_ and O_2_) on either side of the electrodes, and the results were compared against theoretical values. For both half-reactions, the Faradaic efficiency was registered at ∼100% (Figure S9), and the calculated values are elucidated in Tables S2 and S3. The bifunctional nature of the Co–S–B-8 catalyst was substantiated through a comprehensive linear sweep curve spanning from the cathodic region (−1.4 V) to the anodic region (0.8 V) (Figure S10). These findings collectively emphasize the multifaceted excellence of the Co–S–B-8 catalyst in the realm of electrocatalysis for sustainable hydrogen production.

**Figure 8:**
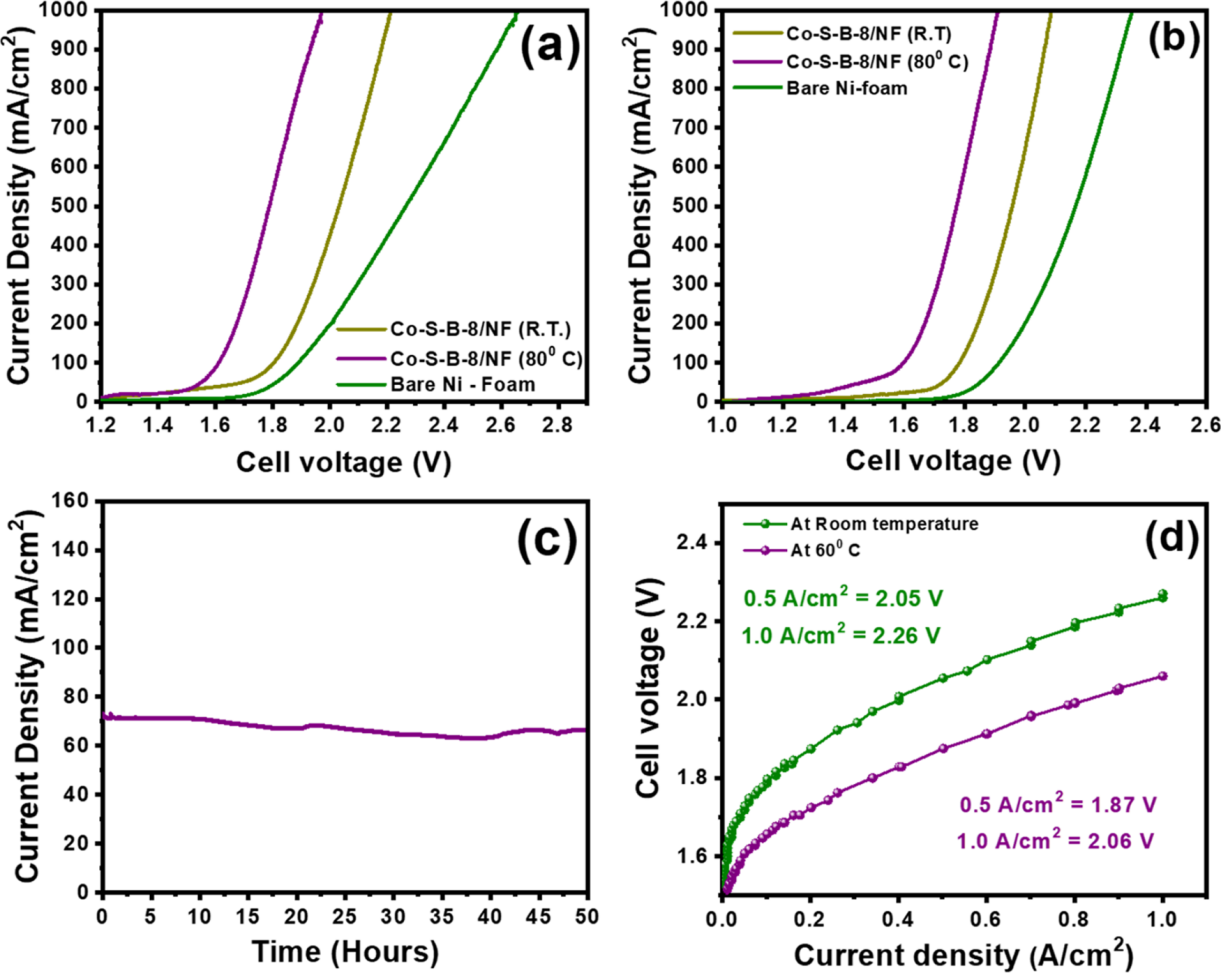
Linear polarization curve recorded in two-electrode configurations (Co–S–B-8/NF || Co–S–B-8/NF) at room temperature (R.T.) and 80 °C in (a) 1 M KOH and (b) 6 M KOH. (c) Long-term stability test in two-electrode configurations under an applied cell voltage of 1.68 V in 6 M KOH. (d) Polarization curve measured for the zero-gap alkaline water electrolyzer (Co–S–B-8/NF || Co–S–B-8/NF) at room temperature and 60 °C in 6 M KOH.

The development of electrocatalysts with robust performance under conditions relevant to industrial settings characterized by high current density and elevated temperature is imperative for commercialization. Thus, the Co–S–B-8/NF catalyst was subjected to comprehensive testing in a conventional electrolysis cell within the two-electrode setup to achieve 1 A/cm^2^ at room temperature (R.T.) and 80 °C. In 1 M KOH aqueous solution, the Co–S–B-8/NF catalyst required a voltage of 2.21 and 1.97 V to achieve 1 A/cm^2^ at room temperature and 80 °C, respectively (Figure 8a). To further demonstrate the relevance for commercial alkaline water electrolyzers that operate under highly alkaline conditions, the Co–S–B-8/NF catalyst was also tested in 6 M KOH at an electrolyte temperature of 80 °C (Figure 8b). Remarkably, the Co–S–B-8/NF catalyst exhibited high activity, demanding 2.08 and 1.91 V to achieve 1 A/cm^2^ at room temperature and 80 °C, respectively. Furthermore, the assembly showcased exceptional stability during a chronoamperometric test at 1.68 V for more than 50 h (Figure 8c). The stability of the Co–S–B-8/NF catalyst was further affirmed by the nearly overlapping polarization curves before and after the 50 h chronoamperometric test (Figure S11). Figure 8d represents the polarization curve (without any IR correction) recorded in a zero-gap single-cell alkaline water electrolyzer (5 cm^2^) using Co–S–B-8/NF || Co–S–B-8/NF as the cathode and anode with 6 M KOH electrolyte. In zero-gap assembly, Co–S–B-8 demonstrates high current densities of 0.5 and 1 A/cm^2^ at low cell voltages of 2.05 and 2.26 V, respectively, at room temperature. The performance improved further when the operating temperature was increased to 60 °C, reaching 1 A/cm^2^ at 2.06 V, corresponding to a voltage efficiency of 71.84%.^42^ This is remarkable for a noble-metal-free electrolyzer setup and is close to other similar reports on noble-metal-free alkaline electrolyzers (Table S6).

## 4 Conclusions

In a nutshell, a unique complex compound was fabricated by combining a metal, a non-metal, and a metalloid in the form of Co–S–B, which presented an amorphous structure and porous 2D morphology. Optimization of the B/S ratio led us to Co–S–B-8 with a B/S molar ratio of 8, showing the best HER and OER rates. The complex compound showed considerably lower HER and OER overpotentials in 1 M KOH compared to Co–B and Co–S catalysts, owing to its lower *R*_ct_, higher intrinsic activity, and higher ECSA, originating from the synergy between the constituents. When tested in a two-electrode assembly, 100 mA/cm^2^ could be attained at a mere 1.77 V with astounding robustness demonstrated through 10,000 cycles and 50 h of stability. In a noble-metal-free zero-gap single-cell electrolyzer comprising Co–S–B on both cathode and anode sides, 1 A/cm^2^ was delivered at 2.06 V at an operating temperature of 60 °C, yielding a voltage efficiency of 71.84%. The work demonstrates a new strategy for preparing electrocatalysts by exploring the combinations of non-metals and metalloids with transition metals, leading the path to discovering high-performing, low-cost electrocatalysts for commercial alkaline water electrolyzers.

## Supporting Information Available

Additional electrochemical data; FE-SEM images; particle size analysis; EDS maps; XPS data; Faradaic efficiency measurements; and comparison tables (PDF)
