# Supplementary material for: Designing Bifunctional Electrocatalysts Based on Complex Cobalt-Sulfo-Boride Compound for High-Current-Density Alkaline Water Electrolysis
Source: Energy Fuels. 2024 Sep 25;38(19):18965–75. doi: 10.1021/acs.energyfuels.4c03171 (PMC11460019; doi:10.1021/acs.energyfuels.4c03171)
Supplement: Supplementary file 1 — ef4c03171_si_001.pdf [file ef4c03171_si_001.pdf]

Supporting Information

for

Designing bifunctional electrocatalyst based on complex Cobalt-Sulpho-Boride compound for high current density alkaline water electrolysis

Akash Suryawanshi[1], Riya Alice B John[1], Aniruddha Bhide[1], Suraj Gupta[2*], Matjaž Spreitzer[2], Rupali Patel[1], Rohan Fernandes[1], Nainesh Patel[1*]

[1]*Department of Physics and Electronics, Christ University, Bengaluru, 560029, India*

[2]*Advanced Materials Department, Jožef Stefan Institute, Jamova 39, 1000 Ljubljana, Slovenia*

***Corresponding authors:**

Dr. Suraj Gupta – suraj.gupta@ijs.si

Prof. Nainesh Patel – nainesh.patel@christuniversity.in

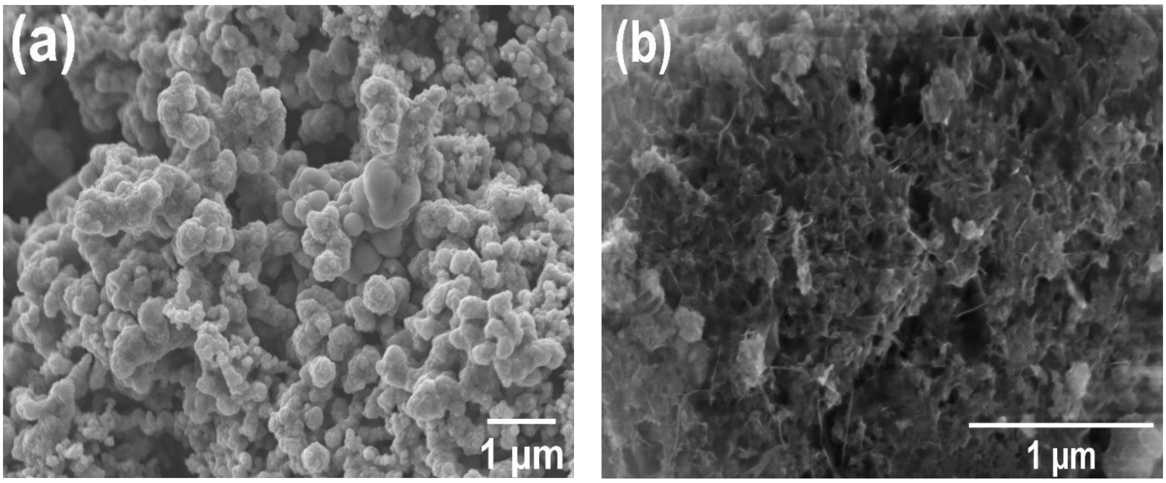

Figure S1: FE-SEM image of a) Co-S; b) Co-S-B-8

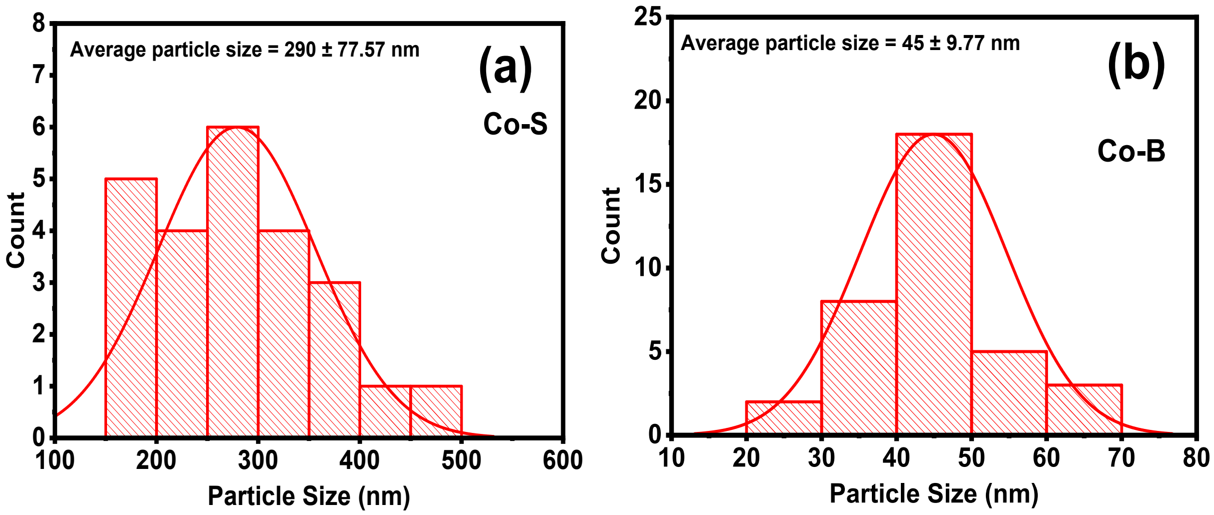

Figure S2: Particle size analysis (a) Co-S; (b) Co-B

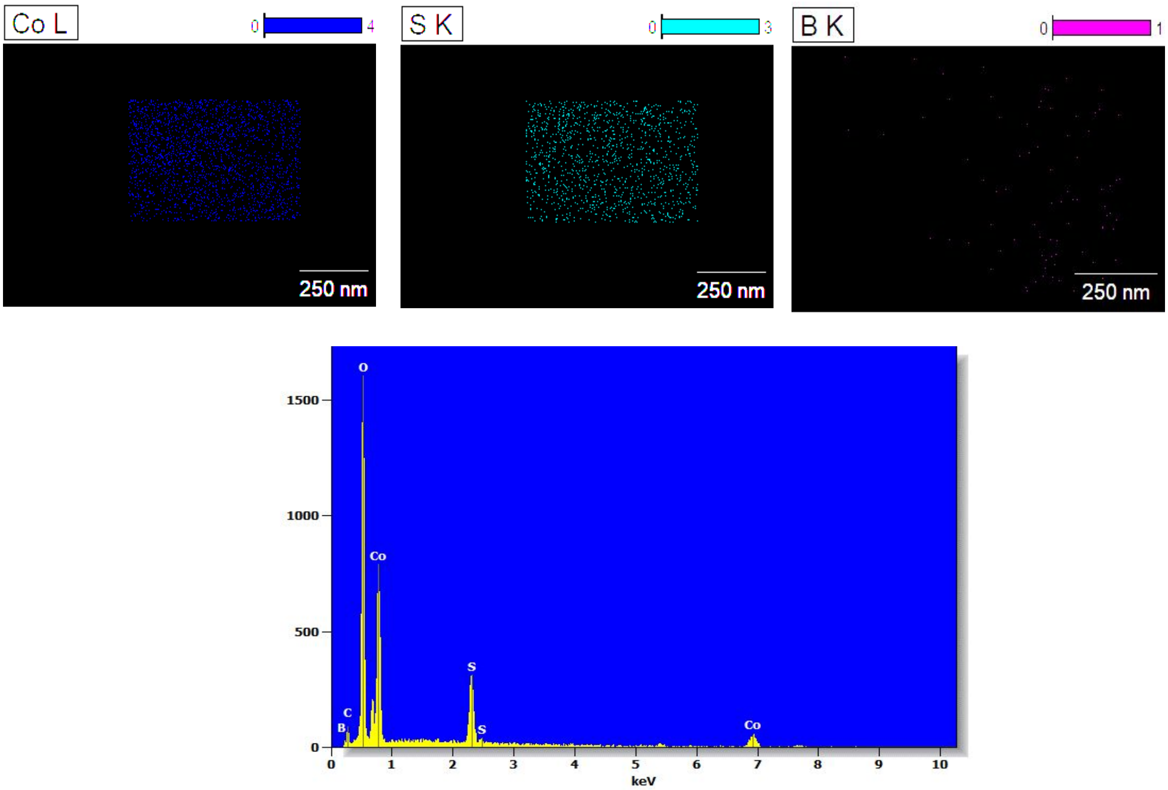

Figure S3: Elemental mapping and EDS profile of Co-S-B-8

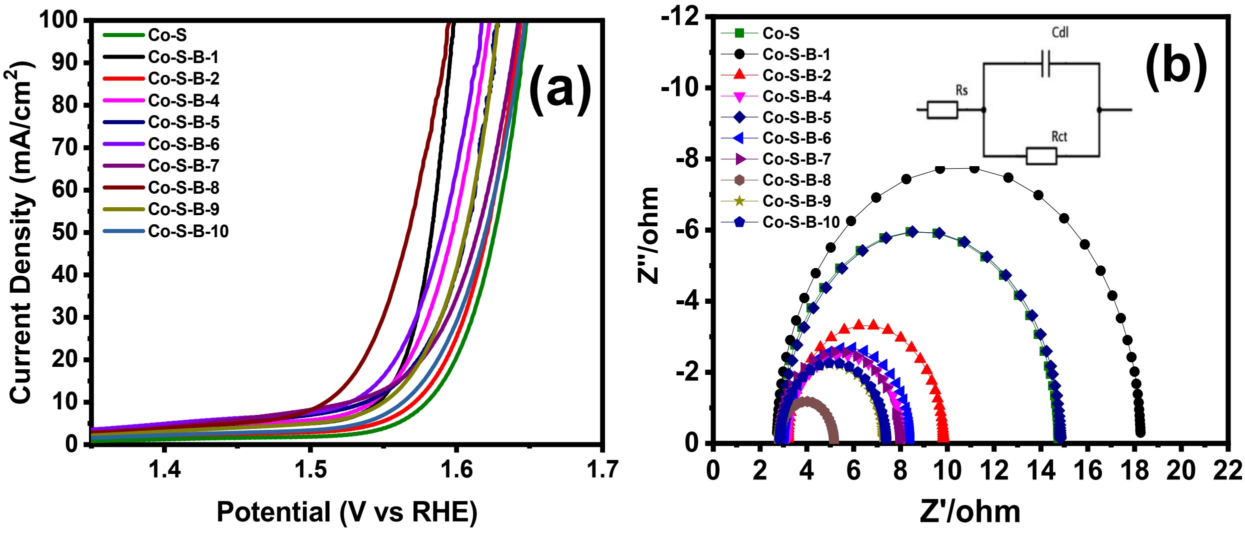

Figure S4: (a) Linear polarization curves (iR compensated) and (b) Nyquist plots for Co-S-B with different B/S ratios.

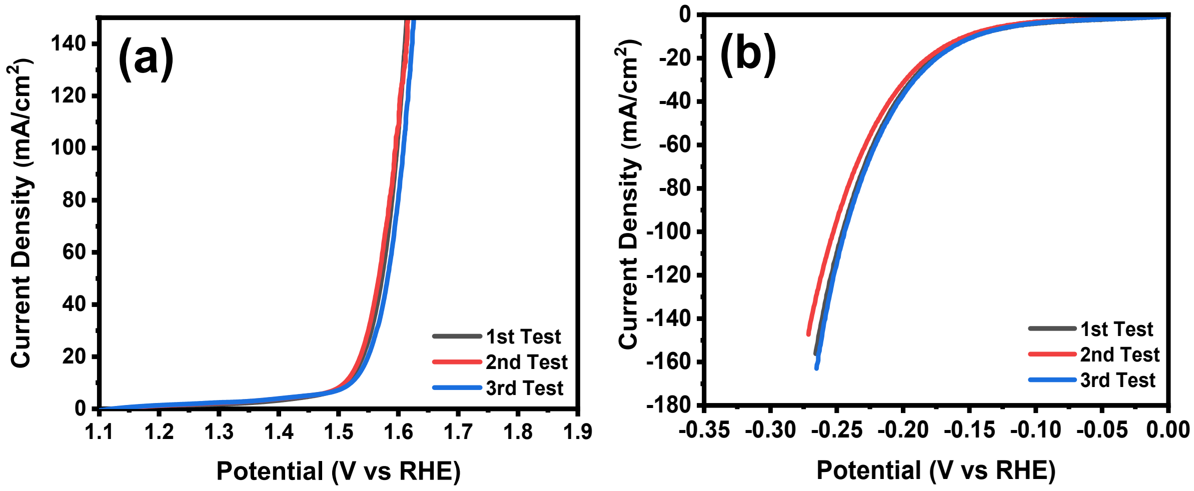

Figure S5; Reproducibility of LSV curve for three different batches of Co-S-B-8 catalyst for (a) OER and (b) HER measured in 1 M KOH.

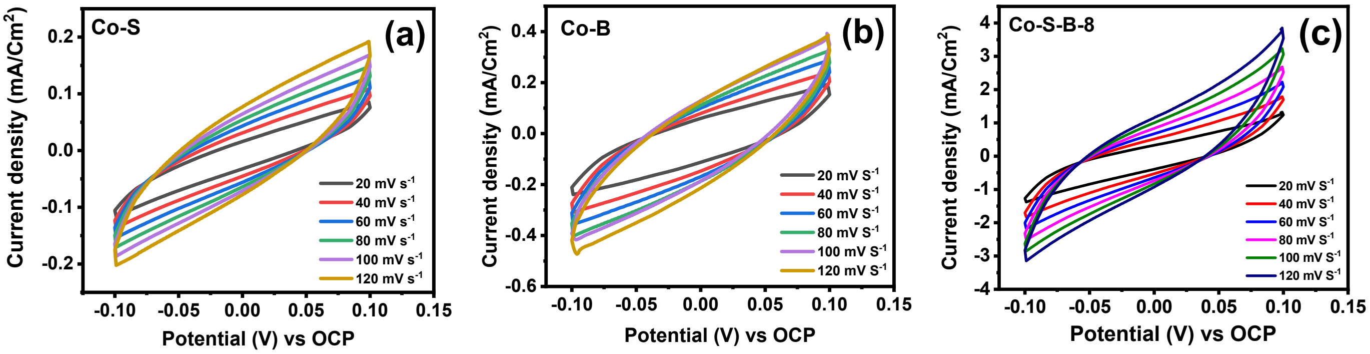

Figure S6. CV curves of (a) Co-S, (b) Co-B, and (c) Co-S-B-8

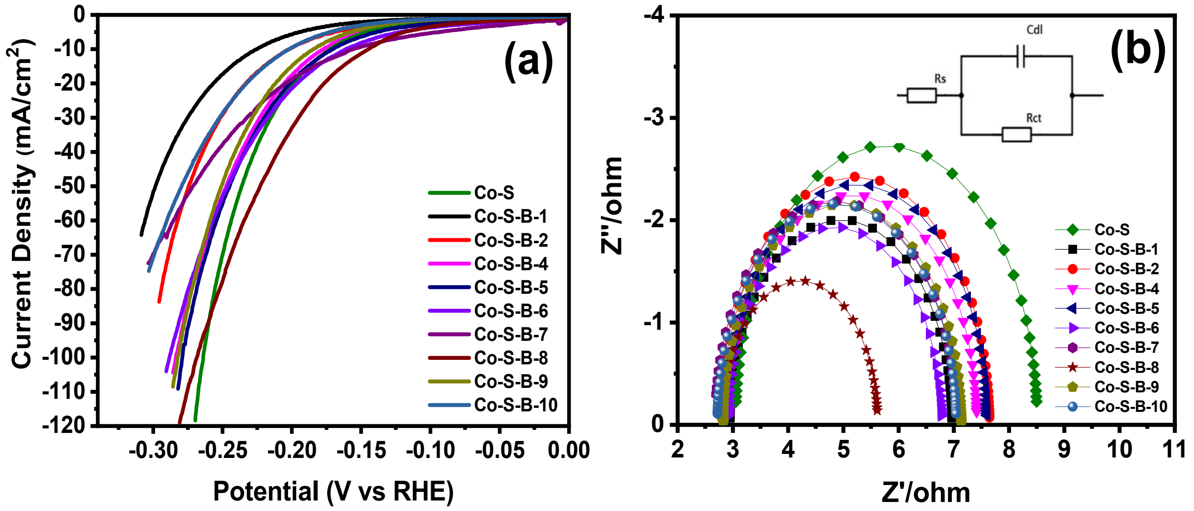

Figure S7: (a) Linear polarization curves (iR compensated) and (b) Nyquist plots for Co-S-B with different B/S ratios.

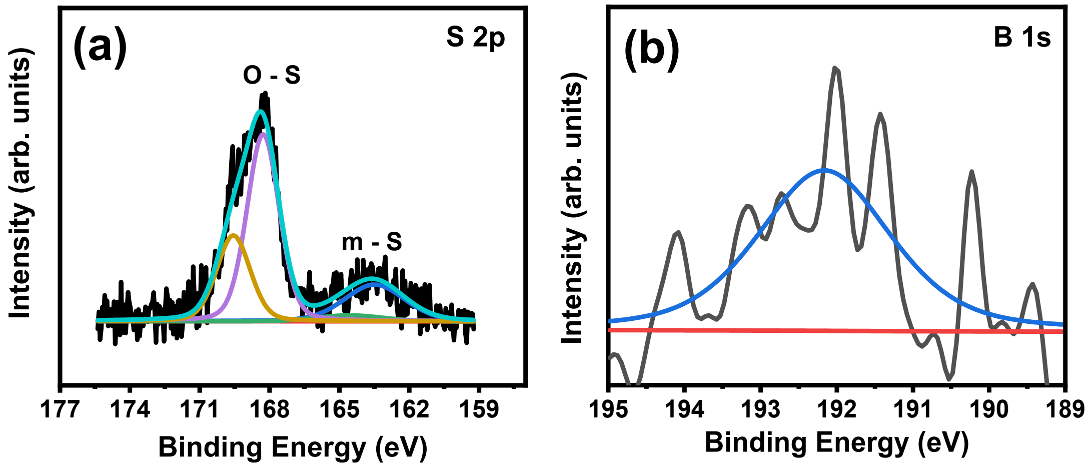

Figure S8: Post-HER high resolution XPS spectra for (a) S2p and (b) B1s states of Co-S-B-8.

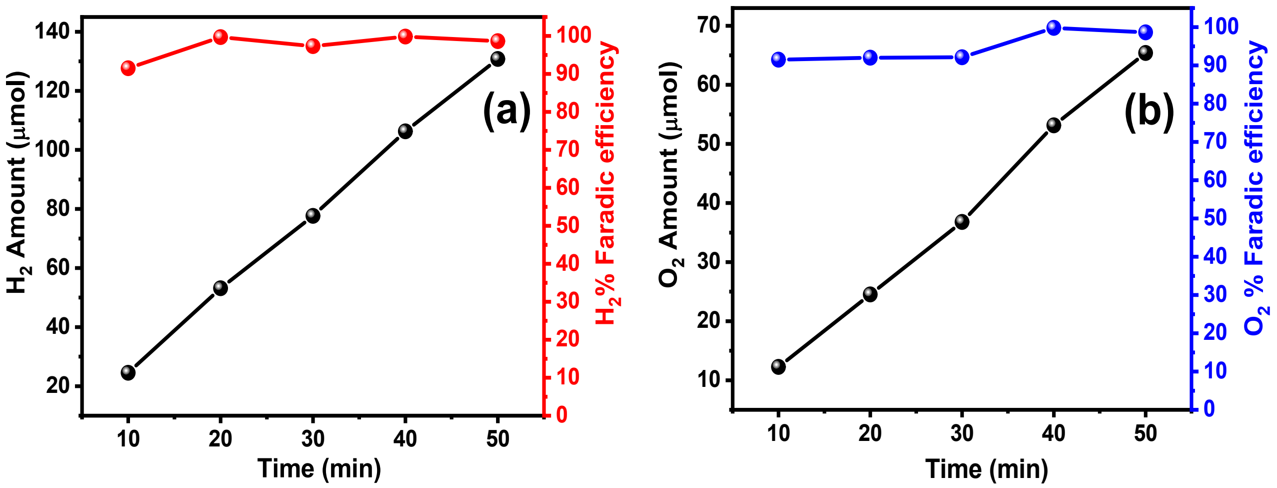

Figure S9: Faradaic efficiency for (a) HER and (b) OER

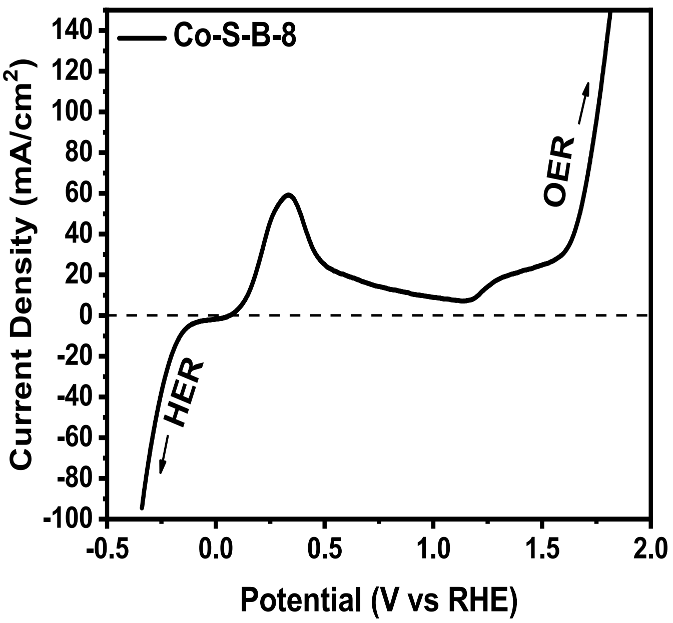

Figure S10: LSV curve for Co-S-B-8 scanned from cathodic region to anodic region showing its ability to work as a bifunctional catalyst in pH 14.

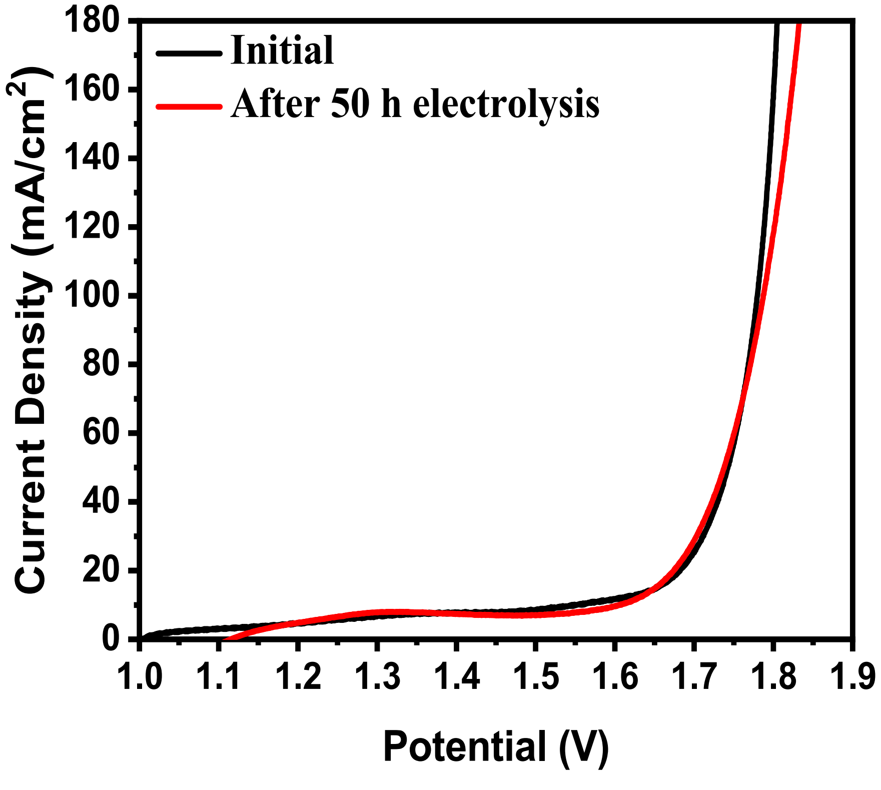

Figure S11: Polarization curves recorded before and after 50 h of electrolysis.

Table S1. Catalyst optimization with different B/S ratios for HER and OER.

Catalyst	HER		OER	
	Overpotential (mV)	R_{ct} (Ω)	Overpotential (mV)	R_{ct} (Ω)
Co-S	172	8.5	330	14.8
Co-S-B-1	225	7.0	316	18.2
Co-S-B-2	202	7.7	340	9.8
Co-S-B-4	177	7.4	312	7.9
Co-S-B-5	170	7.6	307	14.8
Co-S-B-6	162	6.8	298	8.4
Co-S-B-7	155	7.0	297	7.9
Co-S-B-8	144	5.6	280	5.1
Co-S-B-9	184	7.1	320	7.2
Co-S-B-10	202	7.0	333	7.4

Table S2: Faradaic efficiency values for HER

Time (min)	Volume of H_2 gas evolved (mL)	µmol of H_2 evolved (Experimental)	Total charge (Q)	Estimated µmol of H_2 (Theoretical)	Faradaic efficiency % (HER)
10	0.6	24.524	5.172	26.8020	91.50
20	1.3	53.1353	10.284	53.2930	99.70
30	1.9	77.6594	15.408	79.8463	97.31
40	2.6	106.2707	20.544	106.4617	99.84
50	3.2	130.7948	25.59	132.6108	98.63

Table S3: Faradaic efficiency values for OER

Time (min)	Volume of O_2 gas evolved (mL)	µmol of O_2 evolved (Experimental)	Total charge (Q)	Estimated µmol of O_2 (Theoretical)	Faradaic efficiency % (OER)
10	0.3	12.2620	5.172	13.4010	91.50
20	0.6	24.5240	10.284	26.6465	92.03
30	0.9	36.7860	15.408	39.9231	92.14
40	1.3	53.1353	20.544	53.2308	99.82
50	1.6	65.3974	25.59	66.3054	98.63

Table S4: Comparison of OER activity of Co-S-B-8 with state-of-the-art OER electrocatalysts.

Catalyst	Overpotential (η_{10})	Tafel slope	Reference
Co-S-B-8	280	79	This work
Co-W-P-B	262	85.6	[1]
Ni-O	450	96	[2]
NiNiO	279	45	[3]
P-Co_3O_4 NSA	330	72	[4]
FCS@N-CT	330	103	[5]
Co@Co_3O_4	309	51	[6]
Co-P-B	290	42	[7]
Co-W-B	292	78	[8]
CoFeOB	294	-	[9]
$CoWO_4$	389	60	[10]
FeNi-N-CNFs	298	106	[11]
NiFeP	333	69	[12]
NiFe/NiO	245	27.6	[13]

Table S5: Comparison of HER activity of Co-S-B-8 with state-of-the-art HER electrocatalysts.

Catalyst	Overpotential (η_{10})	Tafel slope	Reference
Co-S-B-8	144	83	This work
PVA-Mo_2C/Mo_2N	142	51	[14]
Co-P-B	145	38	[7]
Ni-S-B	240	140	[15]
NS-Mo_2C	210	64	[16]
Co-Ni-B	133	51	[17]
S: CoP/NF	109	54	[18]
M-MoS_2	175	41	[19]
Co-W-B	92	69	[8]
Ru-Ni_2P	132	124	[20]

Table S6: Comparison of electrolyzer performance with state-of-the-art zero-gap alkaline electrolyzers.

Cathode catalyst	Anode catalyst	Electrolyte	Temperature (^{0}C)	Current density and overpotential	Efficiency	Ref.
Co-S-B-8/NF	Co-S-B-8/NF	6 M KOH	60^0 C	1 A/cm^2 @ 2.06 V	71.84%	This work
Pt/C @ carbon paper	Ni-Fe LDH	1 M KOH	55^0 C	1 A/cm^2 @ 1.745 V	--	[21]
Pt/C	IrO_2	1 M KOH	60^0 C	1.2 A/cm^2 @ 2 V	--	[22]
Pt/c	g-CN-CNF-800	1 M KOH	60^0 C	1 A/cm^2 @2 V	--	[23]
$NiMn_2O_4$/CNF	Pt/C	6 M KOH	50^0 C	0.3 A/cm^2 @ 1.8 V	--	[24]
Raney nickel	$NiFe_2O_4$	1 M KOH	60^0 C	0.83 A/cm^2 @ 1.8 V	--	[25]
Ni foam	Ni foam	3 M KOH	60^0 C	0.09 A/cm^2 @ 2 V	--	[26]
NiFeCo	$NiFe_2O_4$	1 M KOH	60^0 C	1 A/cm^2 @ 1.9 V	63.1%	[27]
Pt/C	Ir black	1 M KOH	50^0 C	1 A/cm^2 @ 1.8 V	--	[28]
Ni-Fe	Ni-Fe	1 M KOH	60^0 C	1.2 A/cm^2 @ 2 V	--	[29]
NiMo-NH_3/H_2	Fe-NiMo-NH_3/H_2	1 M KOH	80^0 C	1 A/cm^2 @ 1.57 V	75.1%	[30]
FeNiMo-N_2/H_2	NiMo-N_2H_2	1 M KOH	80^0 C	1 A/cm^2 @ 1.68 V	70.3%	[30]
FeNiMo-NH_3	NiMo-NH_3	1 M KOH	80^0 C	1 A/cm^2 @ 1.62 V	72.6%	[30]
Pt/C	NiFe-LDH	1 M KOH	80^0 C	1 A/cm^2 @ 1.59 V	74.3%	[31]
Pt/C	IrO_x	1 M KOH	60^0C	1 A/cm^2 @ 1.67 V	71.8%	[31]
Pt/C	$Cu_{0.7}Co_{2.3}O_4$	1 M KOH	25^0C	1 A/Cm^2 @ 1.8 V	68.3 %	[32]

References

[1] Bhide, A.; Gupta, S.; Bhabal, R.; Mali, K.; Bhagat, B.; Dashora, A.; Patel, M.; Fernandes, R.; Patel, N. International Journal of Hydrogen Energy Unveiling the synergistic effect of amorphous CoW-phosphoborides for overall alkaline water electrolysis, *Int. J. Hydrogen Energy* **2024**, 63, 645–656.

[2] Arciga-Duran, E.; Meas, Y.; Pérez-Bueno, J. J.; Ballesteros, J. C.; Trejo, G. Effect of oxygen vacancies in electrodeposited NiO towards the oxygen evolution reaction: Role of Ni-Glycine complexes, *Electrochim. Acta* **2018**, 268, 49–58.

[3] Heath, M. M.; Potgieter, M.; Seland, F.; Sunde, S.; Kriek, R. Enhancing the Oxygen Evolution Reaction Activity of Sputtered Ni , NiO , and NiNiO Thin Films by Incorporating Fe, *ChemElectroChem* **2024**, 202300485.

[4] Wang, X.; Li, T. T.; Zheng, Y. Q. Co3O4 nanosheet arrays treated by defect engineering for enhanced electrocatalytic water oxidation," *Int. J. Hydrogen Energy* **2018**, 2009–2017.

[5] Li, J.; Liu, G.; Liu, B.; Min, Z.; Qian, D.; Jiang, J.; Li, J. Fe-doped CoSe2 nanoparticles encapsulated in N-doped bamboo-like carbon nanotubes as an efficient electrocatalyst for oxygen evolution reaction, *Electrochim. Acta* **2018**, 265, 577–585.

[6] Qi, C.; Zhang, L.; Xu, G.; Sun, Z.; Zhao, A.; Jia, D. Co@Co 3 O 4 nanoparticle embedded nitrogen-doped carbon architectures as efficient bicatalysts for oxygen reduction and evolution reactions, *Appl. Surf. Sci.* **2018**, 427, 319–327.

[7] Chunduri, A.; Gupta, S.; Bapat, O.; Bhide, A.; Fernandes, R.; Patel, M.; Bambole,V.; Miotello, A.; Patel, N. A unique amorphous cobalt-phosphide-boride bifunctional electrocatalyst for enhanced alkaline water-splitting, *Appl. Catal. B Environ.* **2019,** 259.

[8] Chunduri, A.; Bhide, A.; Gupta, S.; Mali, K.; Bhagat, B.; Dashora, A.; Spreitzer, M.; Fernandes, R.; Patel, R.; Patel, N. Exploring the Role of Multi-Catalytic Sites in an Amorphous Co-W-B Electrocatalyst for Hydrogen and Oxygen Evolution Reactions, *ACS Appl. Energy Mater.* **2023**, 6, 4630–4641.

[9] Gupta, S.; Forster, M.; Yadav, A.; Cowan, A. J.; Patel, N.; Patel, M. Highly Efficient and Selective Metal Oxy-Boride Electrocatalysts for Oxygen Evolution from Alkali and Saline Solutions, *ACS Appl. Energy Mater.*, vol. 3, no. 8, pp. 7619–7628, 2020, doi: 10.1021/acsaem.0c01040.

[10] Alshehri, S. M.; Ahmed, J.; Ahamad, T.; Arunachalam, P.; Ahmad, T.; Khan, A. Bifunctional electro-catalytic performances of CoWO4 nanocubes for water redox reactions (OER/ORR), *RSC Adv.* **2017,** 72, 45615–45623.

[11] Zhao, X.; Liu, X.; Huang, B.; Wang, P.; Pei, Y. Hydroxyl group modification improves the electrocatalytic ORR and OER activity of graphene supported single and bi-metal atomic catalysts (Ni, Co, and Fe), *J. Mater. Chem. A* **2019**, 7, 24583–24593.

[12] Berger, M.; Popa, I.; Negahdar, L.; Palkovits, S.; Kaufmann, B.; Pilaski, M.; Hoster, H. Palkovits, R. Elucidating the Influence of Intercalated Anions in NiFe LDH on the Electrocatalytic Behavior of OER : A Kinetic Study, *ChemElectroChem* **2023**, 202300235, 1–8.

[13] Jeon, J.; Kim, J.; Kim, T.; Park, C.; Jung, K.; Yoon, J.; Kim, J.; Kim, Y.; Kang, K. Electrochemistry Communications Enhanced oxygen evolution reaction in hierarchical NiFe / NiO electrocatalysts : Effects of electrodeposition condition on electrode, *Electrochem. commun.* **2024**, 160, 107668.

[14] Chen, X.; Qi, J.; Wang, P.; Li, C.; Chen, X.; Liang, C. Polyvinyl alcohol protected Mo2C/Mo2N multicomponent electrocatalysts with controlled morphology for hydrogen evolution reaction in acid and alkaline medium, *Electrochim. Acta* **2018**, 273, 239–247.

[15] Wu, Y.; Gao, Y.; He, H.; Zhang, P. Novel electrocatalyst of nickel sulfide boron coating for hydrogen evolution reaction in alkaline solution, *Appl. Surf. Sci.* **2018**, 480, 689–696.

[16] Wang, D.; Liu, T.; Wang, J.; Wu, Z. N, P (S) Co-doped Mo2C/C hybrid electrocatalysts for improved hydrogen generation, *Carbon N. Y.* **2018**, 139, 845–852.

[17] Gupta, S.; Patel, N.; Fernandes, R.; Kadrekar, R.; Dashora, A.; Yadav, A.; Bhattacharyya, D.; Jha, S.; Miotello, A.; Kothari, D. Co-Ni-B nanocatalyst for efficient hydrogen evolution reaction in wide pH range, *Appl. Catal. B Environ.* **2016**, 192, 126–133.

[18] Ali, M.; Anjum, R.; Okyay, M. S.; Kim, M.; Lee, M. H.; Park, N.; Lee, J. Bifunctional sulfur-doped cobalt phosphide electrocatalyst outperforms all-noble-metal electrocatalysts in alkaline electrolyzer for overall water splitting, *Nano Energy* **2018**, 53, 286-295.

[19] Geng, X.; Sun, W.; Wu, W.; Chen, B.; Hilo, A.; Benamara, M.; Zhu, H.; Watanabe, F.; Cui, J.; Chen, T. Pure and stable metallic phase molybdenum disulfide nanosheets for hydrogen evolution reaction, *Nat. Commun.* **2016**, 7, 1–7.

[20] Chi, J.; Zhang, X.; Ma, X.; Dong, B.; Zhang, J.; Guo, B.; Yang, M.; Wang, L.; Chai, Y.; Liu, C. Interface Charge Engineering of Ultra fi ne Ru/Ni 2 P Nanoparticles Encapsulated in N,P-Codoped Hollow Carbon Nanospheres for E ffi cient Hydrogen Evolution, ACS Sustainable Chem. Eng. **2019**, 21, 17714-17722.

[21] Jiang, W.; Faid, A.; Gomes, B.; Galkina, I.; Xia, L.; Lobo, C.; Desmau, M.; Borowski, P.; Hartmann, H.; Maljusch, A.; Besmehn, A.; Roth, C.; Sunde, S.; Lehnert, W.; Shivro, M. Composition-Dependent Morphology , Structure , and Catalytical Performance of Nickel – Iron Layered Double Hydroxide as Highly-Efficient and Stable Anode Catalyst in Anion Exchange Membrane Water Electrolysis, Adv.

Funct. Mater. **2022**, 32, 2203520.

[22] Gatto, I.; Capri, A.; Vecchio, C.; Zignani, S.; Patti, A.; Baglio, V. Optimal operating conditions evaluation of an anion-exchange-membrane electrolyzer based on FUMASEP ® FAA3-50 membrane, *Int. J. Hydrogen Energy* vol. **2023**, 48, 11914-11921.

[23] Park, J. E.; Kim, M.; Lim, M.; Kang, S.; Kim, J.; Oh, S.; Her, M.; Cho, Y.; Sung, Y. Grapitic carbon nitride-carbon nanofiber as oxygen catalyst in anion exchange membrane water electrolyzer and rechargeable metal-air cells, *Applied Catal. B, Environ.* **2018**, 237, 140-148.

[24] Busacca, C.; Zignani, S.; Blasi, A.; Blasi, O.; Faro, M.; Antonucci, V.; Arico, A. Electrospun NiMn 2 O 4 and NiCo 2 O 4 spinel oxides supported on carbon nanofibers as electrocatalysts for the oxygen evolution reaction in an anion exchange membrane-based electrolysis cell, *Int. J. Hydrogen Energy **2019**,* 4, 20987-20996

[25] Motealleh, B.; Liu, Z.; Masel, R. I.; Sculley, J. P.; Richard, Z.; Meroueh, L. Next-generation anion exchange membrane water electrolyzers operating for commercially relevant lifetimes, *Int. J. Hydrogen Energy* **2020**, 1–8.

[26] Konovalova, A.; Kim, H.; Kim, S.; Lim, A.; Park, H.; Kraglund, M.; Ali, D.; Jang, J.; Kim, H.; Henkensmeier, D. Blend membranes of polybenzimidazole and an anion exchange ionomer (FAA3) for alkaline water electrolysis : Improved alkaline stability and conductivity, *J. Memb. Sci.* **2018**, 564, 653–662.

[27] Liu, Z.; Sajjad, S. D.; Gao, Y.; Yang, H.; Kaczur, J. J.; Masel, R. I. The effect of membrane on an alkaline water electrolyzer, *Int. J. Hydrogen Energy* **2017**, 42, 29661–29665.

[28] Fortin, P.; Khoza, T.; Cao, X.; Yngve, S.; Oyarce, A.; Holdcroft, S. High-performance alkaline water electrolysis using Aemion ™ anion exchange membranes, *J. Power Sources* **2020**, 451, 227814.

[29] Chen, N.; Paek, S.; Lee, J.; Park, J.; Lee, S.; Lee, Y. Environmental Science High-performance anion exchange membrane water electrolyzers with a current density of 7 . 68 A cm^{-2} and a durability of 1000 hours †, Energy Environ. Sci. **2021**, 14, 6338 - 6348.

[30] Chen P.; Hu, X. High-Efficiency Anion Exchange Membrane Water Electrolysis Employing Non-Noble Metal Catalysts, Adv. Energy Mater. **2020**, 2002285.

[31] Koshikawa, H.; Murase, H.; Hayashi, T.; Nakajima, K.; Mashiko, H.; Shiraishi, S.; Tsuji, Y. Single Nanometer-Sized NiFe-Layered Double Hydroxides as Anode Catalyst in Anion Exchange Membrane Water Electrolysis Cell with Energy Conversion Efficiency of 74.7% at 1.0 A cm^{-2}, *ACS Catal.* **2020**, 10, 1886–1893.

[32] Wu X.; Scott, K. CuxCo3-xO4 ($0 \leq x < 1$) nanoparticles for oxygen evolution in high performance alkaline exchange membrane water electrolysers, *J. Mater. Chem.* **2011**, 21, 12344–12351.
